# Membrane distillation for concentrated blackwater: Influence of configuration (air gap, direct contact, vacuum) on selectivity and water productivity

**DOI:** 10.1016/j.seppur.2021.118390

**Published:** 2021-05-15

**Authors:** C.J. Davey, P. Liu, F. Kamranvand, L. Williams, Y. Jiang, A. Parker, S. Tyrrel, E.J. McAdam

**Affiliations:** a Cranfield Water Science Institute, Cranfield University, Bedfordshire MK43 0AL, UK; b GreenTech Environmental Co. Ltd., Wangjing, Chaoyang District, 100102 Beijing, China; c Centre for Creative and Competitive Design, Cranfield University, Bedfordshire MK43 0AL, UK; d Centre for Thermal Energy Systems and Materials, Cranfield University, Bedfordshire MK43 0AL, UK

**Keywords:** Membrane distillation, Air gap, Direct contact, Vacuum, Urine, Faeces, Sanitation, Wastewater

## Abstract

Water recovery from concentrated blackwater has been studied using air gap (AGMD), direct contact (DCMD) and vacuum membrane distillation (VMD) to deliver decentralised sanitation. Whilst good water quality was achieved with each configuration, differences in the rejection of volatile compounds was observed. VMD exhibited the highest rejection of volatiles, specifically ammoniacal nitrogen, of all the configurations but fouling inhibited total flux. DCMD exhibited a temperature dependent volatile rejection which resulted in poor rejection at lower feed temperatures (≤40 °C). AGMD was identified as the most promising configuration for application within decentralised sanitation, since the rejection of volatiles was consistent over a range of operating temperatures with ammonia rejection directly related to solution pH. An increase in organic colloids and particles due to faecal contamination reduced COD removal due to the induction of wetting, but was shown to be offset by adoption of a smaller pore size (0.1 μm), and when complemented with upstream solid-liquid separation within a fully integrated system, will provide a robust sanitation solution. Importantly, this work has shown that AGMD can recover water from concentrated blackwater close to international discharge and reuse regulations in a single stage process; this is significant as blackwater consists of only urine and faeces, and is thus 40 times more concentrated than municipal sewage. It is proposed that the water quality produced reflects a step change to delivering safe sanitation, and is complemented by a simple method for heat recovery integration this is similarly advantageous for resource constrained environments common to decentralised sanitation solutions.

## Highlights

• Membrane distillation configuration studied for water recovery from blackwater.
• Selectivity altered by differences in vapour pressure and water flux.
• Wetting by organics limited by smaller pore size but decreases mass transfer.
• Organics fouling more severe for vacuum configuration.
• Air gap provides more robust separation over temperature range studied.

## 1 Introduction

Bringing about improved sanitation to the 2.4 billion people globally who currently lack access [1] requires huge economic investment estimated at around US$136.5 billion per year to establish the centralised networks and wastewater treatment facilities required [2]. However, direct implementation of available advanced technologies within a low income country context is an unsustainable approach, as they do not account for the specific needs and constraints of the low resource settings [3]. Electrical power can be intermittent, unreliable or non-existent, so technologies must make use of off-grid and alternative energy sources. Similarly, the availability of fresh water is limited so the use of flush water must be avoided [4], resulting in a liquid waste significantly more concentrated than traditional blackwater and consisting almost entirely of urine and faeces. To discharge the water recovered from this mixture into the environment or reuse it for agricultural irrigation a huge reduction by several orders of magnitude of its nitrogen, organic and pathogenic content should be achieved to meet current international guidelines (e.g. US EPA, WHO, EU 91/271/EEC) [5], [6], [7], [8] if a transition toward safe sanitation is to be achieved. Therefore, an enormous load is placed on any single treatment technology for water recovery from this “concentrated blackwater” and simple, efficient, novel technologies are required to provide a sustainable and economical pathway to improved sanitation.

In such circumstances, thermal separations such as distillation which exploit the difference in volatilities of the components of a mixture can be advantageous when compared to alternative separation techniques [9]. A purely physical and highly selective separation can be achieved, relatively unimpeded by increasing solution concentration and with potential to recover water to regulatory standards. Energy consumption is dictated by volumetric flow rather than pollutant concentration, which is attractive for low volume concentrated wastewater treatment (~1.55 L cap^-1^ d^-1^: Urine & Faeces [10]). The thermal energy required is also generally accessible and reliable throughout many developing countries evidenced by the widespread use of solid fuel cookstoves [11] but also with potential to be harnessed through solar thermal or biogas.

**Fig. 1:**
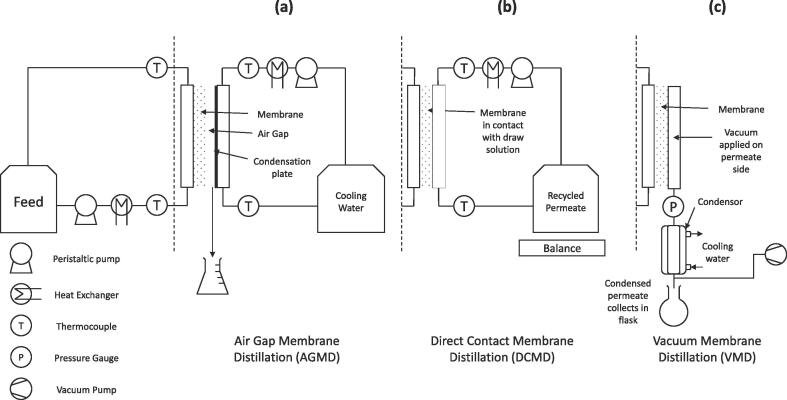
Schematics of the configurations of the different membrane distillation processes studied showing the different permeate side configurations for (a) AGMD with an aluminium condenser plate and recirculating cooling water, (b) DCMD where the cooled draw solution is in direct contact with the permeate side of the membrane and (c) VMD where vacuum is applied on the permeate side of the membrane.

To increase energy efficiency and reduce the overall capital requirement for distillation, a microporous hydrophobic membrane can be used. A heated feed is brought into contact with one side of the membrane and the hydrophobicity of the material prevents liquid from entering the pores which replace the vapour space of a distillation column as each of the micrometre sized pores supports a liquid-vapour interface [12]. The resulting evaporative surface area to volume ratio is incredibly high, avoiding the need to heat the liquid mixture to its boiling temperature, whilst simultaneously miniaturising the overall process size. To facilitate membrane distillation and induce the required vapour pressure difference across the membrane, a number of configurations exhibiting specific trade-offs in terms of thermal efficiency and mass transfer can be utilised. The three most commonly studied configurations for water recovery include air gap membrane distillation (AGMD; Fig. 1a), direct contact membrane distillation (DCMD; Fig. 1b) and vacuum membrane distillation (VMD, Fig. 1c) [12], [13]. For AGMD, a cooling plate is set a defined distance away from the permeate side of the membrane and used to condense the permeating vapour. The insulating air gap limits conductive heat loss through the membrane, therefore generally exhibiting high energy efficiency, but also imposes an additional resistance to mass transfer, so a lower productivity is observed [13]. In DCMD a cooled draw solution is contacted with the permeate side of the membrane enhancing the mass transfer (higher productivity) but is coupled by an increased conductive heat transfer (lowering energy efficiency) [14]. With VMD, a vacuum is applied on the permeate side of the membrane, mass transfer is enhanced by removing vapour from the pores of the membrane, decreasing the thermal input for evaporation, and there is negligible conductive heat transfer through the membrane. Due to the large hydrostatic pressure difference across the membrane, it can be more susceptible to fouling and wetting of its pores [15], [16]. In addition, the difficulty in scaling down high vacuum pumps to the required process scale and energy requirements make the transition to decentralised sanitation extremely challenging.

For desalination, which remains the most common application of membrane distillation studied, several investigations have compared AGMD, DCMD and VMD. A highly selective separation is observed by all configurations due to the sole presence of non-volatile salts, mainly sodium chloride, and as such each configuration is being considered at commercial scale [17], [18], [19], [20]. Comparative assessments have subsequently centred on identifying the most energy efficient configuration and operating conditions [21], [22], [23], [24]. In contrast, when applied to the recovery of volatile solutes, such as ammonia or volatile organic compounds, it is recognised that the selectivity of different membrane distillation configurations exhibit large variation dependent upon the specific operating conditions arising from the way the vapour pressure gradient is sustained [16], [25], [26], [27], [28], [29], [30], [31], [32]. Therefore, when membrane distillation is applied for water recovery from a complex matrix comprised of volatile and non-volatile organic and inorganic constituents, such as concentrated blackwater, configuration selection can be regarded as critical to achieving the necessary selectivity in water separation. Importantly, the application of membrane distillation for water recovery from urine [33], [34], [35], [36] has been successfully demonstrated on several space missions [37], evidencing resilience in analogous conditions. Nevertheless, the presence of various organic and inorganic solutes can also increase the propensity for membrane wetting, through either lowering of the feed solution surface tension or decreasing the membrane hydrophobicity through surface fouling, with each of these effects being exacerbated to varying degrees with different configurations [38]. Identification of the optimal configuration can limit the effect of these hindrances and help address the pressing need for advanced solutions in decentralised sanitation.

Therefore, this investigation has studied the separation performance of the three main membrane distillation configurations (AGMD, DCMD, VMD) for water recovery from concentrated blackwater, to establish the technological potential for application to decentralised sanitation. The specific objectives were to: (i) compare membrane distillation configurations to determine differences in separation behaviour, with further emphasis on selectivity, (ii) determined the distinction in the separation mechanisms provided by different configurations through challenging with critical operational variables, and (iii) demonstrate whether the preferred configuration can facilitate safe pathogen free water recovery from concentrated blackwater when exposed to elevated levels of faecal contamination to ascertain the process robustness and help inform on the overall process design needed to deliver safe and effective decentralised sanitation.

## 2 Materials and methods

### 2.1 Experimental set up

The experimental set-up used was adaptable to either AGMD, DCMD or VMD (Fig. 1) using the same acrylic membrane distillation cell (1160071, Sterlitech, Kent WA, USA) which exposed a membrane surface area of 0.014 m^2^. Unsupported hydrophobic symmetric polytetrafluoroethylene (PTFE) membranes (Cobetter Filter, Hangzhou, China) exhibiting different manufacturer stated nominal pore sizes of 0.1, 0.45, 1, 3 and 5 μm were used in all experiments (Table 1). The feed side consisted of a recirculating peristaltic pump (520S, Watson Marlow, Falmouth UK) and stainless steel heat exchanger immersed in a water bath (TC120, Grant, Devizes UK) to achieve a feed inlet temperature of 30, 40, 50 or 60 °C and was the same for each configuration. The cross flow velocity through the MD module was kept constant at 0.12 m s^−1^ for all experiments. For AGMD and DCMD the permeate side consisted of a peristaltic pump (530S, Watson Marlow, Falmouth UK) recirculating deionised water through a stainless steel heat exchanger immersed in a heater chiller (GD120, Grant, Devizes UK) to achieve a temperature of 20 °C at the inlet to the membrane module and passing either behind an aluminium cooling plate producing an air gap of 5.6 mm (AGMD) or in direct contact with the membrane (DCMD). For VMD, a vacuum (≤45 mbar absolute) was applied to the permeate side of the membrane with a vacuum pump (MZ 1C, Vacuubrand, Wertheim DE) and measured with a pressure gauge (PXM319-001A10V, OMEGA, Manchester UK). Permeate vapour was condensed in a glass vacuum jacketed condenser (Radleys, Saffron Walden UK) cooled to 2 °C using a recirculating chiller (LT Ecocool 150, Grant, Devizes UK) and collected in a glass round bottomed flask. Combined conductivity / temperature data loggers (CDH-SD1, OMEGA, Manchester UK) were placed in-line at the inlet and outlets of the membrane distillation cell to record the temperature differential.

**Table 1:** Membrane material properties.

	Manufacturer stated nominal pore size (μm)				
	0.1	0.45	1	3	5
Measured Porosity (%)*	80.8 (±1.66)	83.9 (±0.82)	85.9 (±1.3)	87.3 (±1.9)	88.2 (±0.65)
Manufacturer Stated Water LEP (bar)	5	2.3	0.66	0.58	0.34
Manufacturer Stated Thickness (μm)	49	51	99	77	47

*Porosity measured according to the method described by Smolders & Franken [39], with observed values typical for commercial PTFE membranes [40].

### 2.2 Analytical methods

Discrete samples of the feed and permeate were taken at defined time intervals (1 h) over the course of the experiments (5 h) to measure flux and water quality. Solution pH (4330, Jenway, Stone, UK) and conductivity (CDH-SD1, OMEGA, Manchester UK) of the discrete samples were determined using the appropriate probes calibrated to standard solutions (pH 4, 7, 10, Fisher Scientific, Loughborough UK; 1500 μS cm^−1^, ±1%, @ 25 °C, OMEGA, Manchester UK). Chemical oxygen demand (COD) and ammoniacal nitrogen were analysed using commercial photometric methods (Spectroquant® cell tests, 11451 COD, 114558 NH_4_-N, Merck, Watford UK) according to the manufacturer’s instructions. Analysis of viable *Escherichia coli* (*E. coli*) was conducted through enumeration and counting of the number of colony forming units (CFU) using standard methods 9215C (spread plate method) and 9215D (membrane filter method) (APHA/AWWA/WEF, 2012) to cover the corresponding concentration ranges of the feed and permeate respectively. The limit of detection (LOD) was 1 log_10_ CFU mL^−1^ and −1 log_10_ CFU mL^−1^ for the spread plate and membrane filter methods respectively. Brilliance *E. coli* agar (CM1046B, ThermoFisher, Loughborough UK) was used and prepared according to the manufacturer’s instructions. Maximum recovery diluent (CM0733, ThermoFisher, Loughborough UK) was used for serial dilutions and pre-wetting / rinsing the membrane filter units (EZ Fit Filtration Unit, 0.22 μm, Merck, Watford UK).

When calculating rejection for DCMD the dilution of the permeating vapours by the draw solution must be taken into account to be able to compare with VMD and AGMD [41]:

(1) $$C_{a,P}=\frac{V_{D,n}C_{a,D.n}-{V_{D,n-1}C}_{a,D.n-1}}{V_{D,n}-V_{D,n-1}}$$

where *C_a,P_* is the concentration of species *a* in the permeate, *V_D,n_* is the volume of the draw solution at time n, *C_a,D,n_* is the concentration of species *a* in the draw solution at time *n*, *V_D,n-1_* is the volume of the draw solution at time *n* − *1* and *C_a,D,n-1_* is the concentration of species a in the draw solution at time *n* − 1.

The observed rejection of a particular component (*R_j,a_*) by each of the membrane distillation configurations can therefore be calculated using:

(2) $$R_{j,a}=\left(1-\frac{C_{a,p}}{C_{a,f}}\right)\times 100\%$$

where *C_a,p_* refers to the concentration of species a in the permeate (where the dilution of permeating vapours in DCMD have been accounted for using Eq. (1)) and *C_a,f_* is the concentration of species a in the feed.

The Log10 Reduction of CFU between the feed and permeate was calculated using:

(3) $${\mathrm{Log}}_{10}\;Reduction\;CFU={\mathrm{Log}}_{10}\left(\frac{C_{f}}{C_{p}}\right)$$

where *C_f_* and *C_p_* are the concentration of CFU in the feed and permeate respectively, with the dilution of permeate in the DCMD configuration accounted for using Eq. (1).

**Fig. 2:**
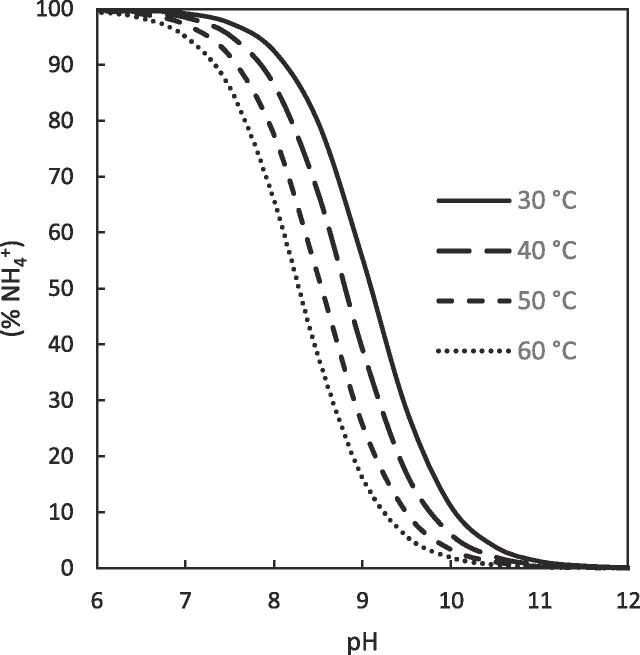
Estimated equilibrium between ammonium and ammonia as a function of the pH and temperature.

The ratio of ammonium to ammonia in solution is a function of the solution pH and temperature (Fig. 2) and can therefore be predicted from the dissociation constants [42] according to the equilibrium:

(4) $${\mathrm{NH}}_{3}+H_{2}O⇌{\mathrm{NH}}_{4}^{+}+{\mathrm{OH}}^{-}$$

where a change in solution pH shifts the equilibrium around the p*K*_a_, whereas a change in the solution temperature shifts the species p*K*_a_.

### 2.3 Materials

Urine and faeces were collected daily with informed consent obtained from each donor and following an anonymous procedure approved through the Cranfield University Research Ethics System (CURES: 2310/207; 2407/2017). Urine was collected in polypropylene pots and the individual samples mixed and either used immediately or stored for a maximum of 18 h at <5 °C. Faecal samples were used immediately after collection. If required, urine samples were allowed to warm to room temperature (23 °C ± 2) before addition to faeces at the desired gravimetric ratio (Faeces/Urine (wt%*)*, 7, 1.8, 0.4) with ~7 wt% representative of average human production [10]. The mixture was thoroughly stirred for 15 min at 400 rpm using an overhead stirrer (RZR 2021, Heidolph, Schwabach DE) followed by coarse filtration with a 2 mm sieve to remove large particles and prevent pipe clogging within the experimental set-up.

## 3 Results and discussion

### 3.1 Membrane distillation can produce high quality water from concentrated blackwater but volatile rejection varies for each configuration

**Fig. 3:**
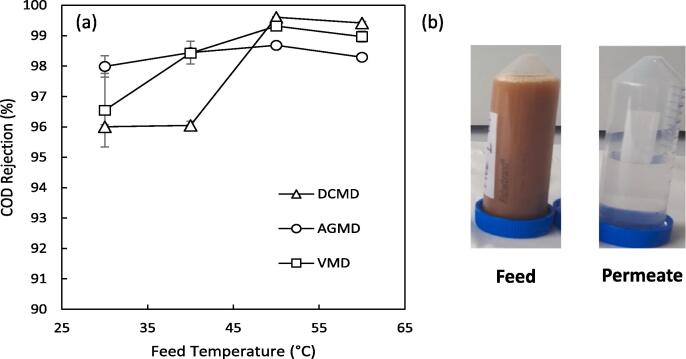
(a) Comparison of COD rejection at different temperatures by AGMD, DCMD and VMD (b) photos showing representative feed and permeate quality. (T_p,i_ = 20 °C for DCMD and AGMD, 0.1 μm PTFE membrane; 1.8 wt% (Faeces/Urine) pH: ~7–9, COD: 7–15 g L^–1^, NH_4_-N: 0.1–1.3 g L^–1^, E. coli: ~4–8 log_10_ CFU mL^−1^).

Initially comparison has been made between AGMD, DCMD and VMD at differing feed temperatures for the selective separation of water from the organic fraction of concentrated blackwater (Fig. 3a). A huge reduction in COD from 7–15 g L^–1^ to 0.054–0.2 g L^–1^ was achieved from the highly concentrated feed consisting solely of urine and faeces due to the majority of the organic fraction being composed of non-volatile compounds such as urea. For DCMD and VMD there is a small increase (3–4%) in the rejection of COD with an increase in temperature (30–60 °C) whereas AGMD exhibits a relatively consistent COD rejection (98% ± 0.25). The temperature dependent rejection indicates that this is due to the vapour phase equilibrium across the membrane as opposed to wetting from feed solution chemistry. High concentrations of organic compounds have been shown to induce membrane wetting through either membrane surface fouling [43] or altering solution surface tension [36]. Both of these effects cause a reduction in the capillary pressure which supports the gas phase within the pores, eventually allowing for liquid bridging through the hydrophobic membranes pores [38] but in this case, can be discounted due to a consistent feed composition across configurations and temperatures. Additionally, an increase in temperature would most likely decrease solution surface tension and therefore reduce rejection at higher temperatures [38], which is contrary to the observed trend. In this study, the permeating organic fraction most likely consisted of primarily volatile organic compounds, rather than non-volatile organic contamination introduced through membrane wetting due to the consistently high organics rejection (Fig. 3a,b), and increased rejection at higher temperatures for DCMD and VMD. Several studies have reported increased mass transfer of, but decreased selectivity towards, volatile organic compounds (VOCs) by VMD and DCMD at increasing feed temperatures due to proportionally larger increases in water transport [27], [44]. For example, in the recovery and purification of ethanol from fermentation broths it is known for both VMD [45] and DCMD [31], an increase in feed temperature increases the mass transfer of water relative to ethanol due to a comparatively higher increase in the saturation vapour pressure of water, resulting in a decreased selectivity towards ethanol. In contrast, AGMD has been demonstrated to generally exhibit a consistent separation factor with an increase in temperature [30], as demonstrated in this study, due to the saturated stagnant air gap which limits the vapour pressure driving force of the minor component (VOCs) at lower temperatures compared to DCMD and VMD. The difference in selectivity is therefore an artefact of how each configuration induces the required vapour pressure gradient.

**Fig. 4:**
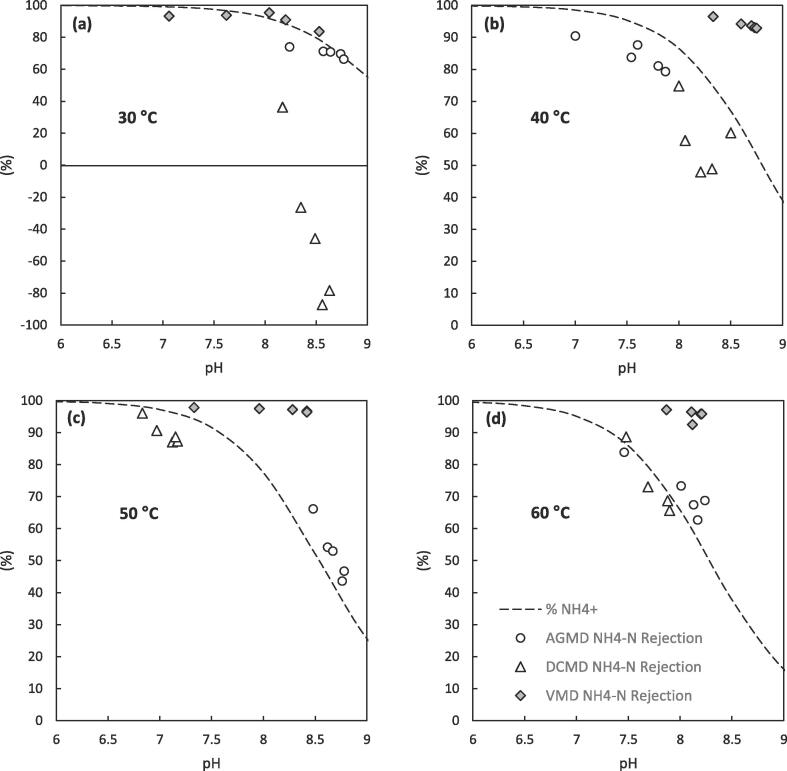
Rejection of ammonia by each configuration at (a) 30 °C, (b) 40 °C, (c) 50 °C and (d) 60 °C showing the % of N present as ammonium in the liquid phase as guide for expected rejection in vapour phase, (T_p,i_ = 20 °C, 0.1 μm PTFE, 1.8 wt% (Faeces/Urine) pH: ~7–9, COD: 7–15 g L^–1^, NH_4_-N: 0.1–1.3 g L^–1^, E. coli: ~4–8 log_10_ CFU mL^−1^).

This selectivity was further demonstrated through the rejection of ammonia, which represents a volatile compound within concentrated blackwater and a heavily regulated contaminant and therefore a critical determining factor for the feasibility of the separation. The rejection is partly determined in each configuration by the ammonia ⇌ ammonium equilibrium (Eq. (4)) which is dependent on solution pH and temperature, as ammonia is volatile and able to permeate across the vapour space within the membrane pores, whereas the ammonium ion is not [46]. The evolution of free ammonia from the hydrolysis of amide functional groups present in the organic fraction of concentrated blackwater (predominantly urea) also leads to an increase in total concentration and solution pH throughout the experiments [47]. For AGMD the rejection of ammoniacal nitrogen can be related directly to the fraction of ionised ammonium (NH_4_^+^) in solution at all temperatures (Fig. 4). This reflects the results observed for COD rejection where the rejection of volatile compounds by the AGMD process is largely unaffected by a change in the feed temperature (Fig. 3a). In contrast, the rejection of ammoniacal nitrogen by DCMD and VMD deviates from the liquid phase ammonia ⇌ ammonium equilibrium with a temperature dependence. For DCMD the ammoniacal nitrogen rejection decreases far below the fraction of ionised ammonium in solution at lower feed temperatures (≤40 °C), with a negative rejection observed at 30 °C. In contrast, at all temperatures studied ammoniacal nitrogen rejection by VMD is > 92% (Fig. 4). Previous studies have evidenced the relationship between temperature and ammonia removal for DCMD and VMD; however, in these studies the emphasis was on ammonia recovery and the feed pH was generally kept high (~pH 10) ensuring sufficient free ammonia in solution to enhance its removal [25]. Several examples have shown for solution pH below 9 with VMD, ammonia mass transfer decreases which also reduces selectivity toward ammonia (i.e. higher rejection) [25], [47]. Ammonia mass transfer in AGMD on the other hand has been largely disregarded in the literature. Of the few studies reported, a reduction in solution pH (i.e. acidification) has been utilised to ensure the equilibrium is shifted towards NH_4_^+^ and a high ammoniacal nitrogen rejection ensured (>99%) [48]. Although this approach could be utilised within each configuration to ensure a high rejection of ammonia, the associated chemical demand required for continuous pH adjustment can be incredibly high. This would add a large additional operational cost to the process, inhibitive for the application of a decentralised sanitation solution within a low resource setting. Alternatively, ammonia separation could be controlled through understanding and control of the precise vapour-liquid equilibrium of membrane distillation.

**Fig. 5:**
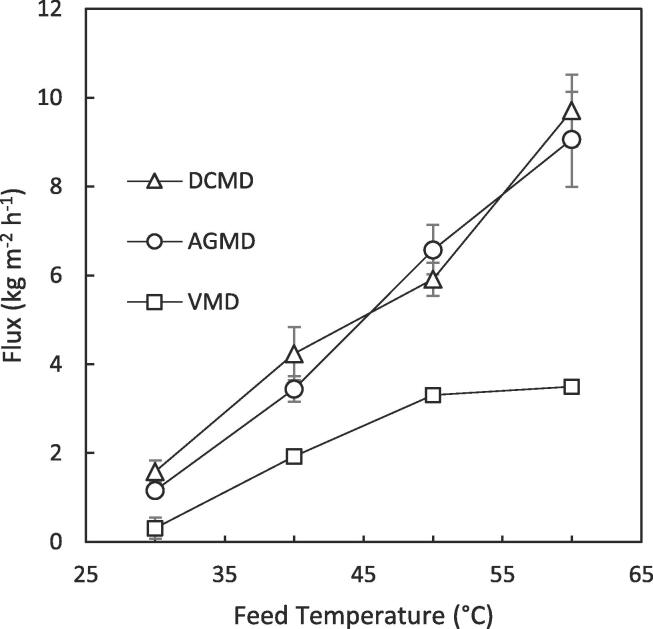
Comparison of total flux of AGMD, DCMD and VMD at varying feed temperatures; (T_p,i_ = 20 °C for DCMD and AGMD, 0.1 μm PTFE membrane; 1.8 wt% (Faeces/Urine) pH: ~7–9, COD: 7–15 g L^–1^, NH_4_-N: 0.1–1.3 g L^–1^, E. coli: ~4–8 log_10_ CFU mL^−1^).

The discrepancy between the separation performance of each configuration can be further described by a combination of the driving force for mass transfer of a volatile species a and the relative water flux. The transmembrane flux of species a is proportional to its partial vapour pressure difference across the membrane [25], [30], [47]:

(5) $$N_{a}=k\left(P_{a,f}-P_{a,p}\right)$$

where $N_{a}$ is the transmembrane flux of component a, k is the permeability of the membrane, $P_{a,f}$ / $P_{a,p}$ are the partial vapour pressures of component a in the feed and at the permeate side of the membrane respectively and the partial pressure of species a ($P_{a})$ is proportional to its molar concentration. For DCMD and VMD partial vapour pressure of species a at the permeate side of the membrane is relatively very low due to either deionised water cooling fluid with low concentration of species a (DCMD) or a vacuum removing all molecules from the permeate side membrane surface (VMD). For this reason, permeate side mass transfer resistance is generally ignored for ammonia in these processes [25], [47], [49]. This was evidenced for DCMD by the comparatively larger ammonia flux due to the low concentration in the permeate cooling stream, which is demonstrated through its negative rejection at 30 °C (Fig. 4a). The ammonia flux increases marginally with temperature between 30 and 60 °C [25], compared to the more considerable increase in water flux (~1.6 to 9.7 kg m^–2^h^−1^; Fig. 5) thereby diluting ammonia in the permeate resulting in an enhanced observed rejection (Fig. 4d). For AGMD on the other hand the partial vapour pressure of species a in the stagnant air gap at the permeate side of the membrane will only be partially lower than the membrane pores, which will be directly related to the partial vapour pressure in solution increasing the resistance to mass transfer [30]. For ammonia, the partial pressure in the feed solution will reflect that in the air gap. This low concentration difference imposes negligible flux and approached equilibrium with the concentration of free ammonia in the feed. For VMD, the vacuum pressure (~45 mbar) is equivalent to the vapour pressure of water at 30 °C indicating that the relative water flux will be much greater than the ammonia flux at all temperatures, observed by a high ammonia rejection in all circumstances [47].

**Fig. 6:**
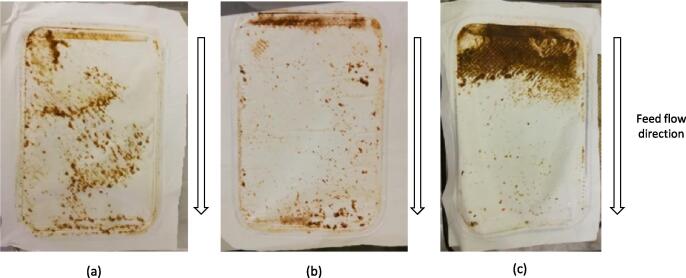
Pictures of the feed side of the membrane (a) AGMD, (b) DCMD and (c) VMD (T_p,i_ = 20 °C for DCMD and AGMD, 0.1 μm PTFE membrane; 1.8 wt% (Faeces/Urine) pH: ~7–9, COD: 7–15 g L^–1^, NH_4_-N: 0.1–1.3 g L^–1^, E. coli: ~4–8 log_10_ CFU mL^−1^).

**Fig. 7:**
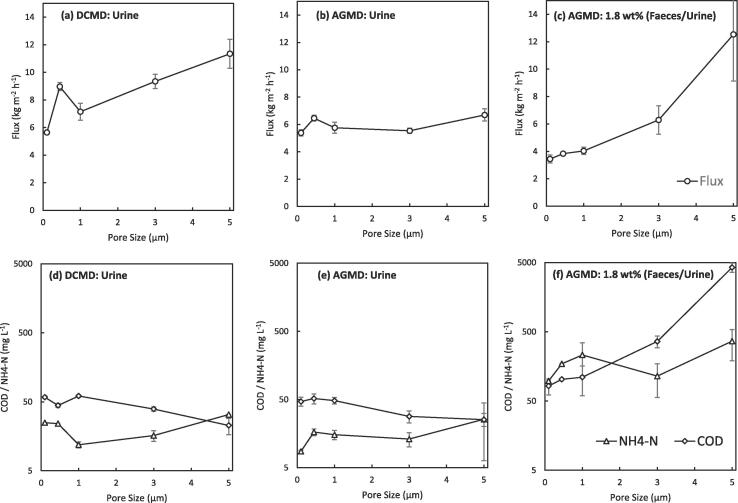
Effect of pore size on the recovery of water from urine and concentrated blackwater by DCMD and AGMD with (a,b,c) total flux, (d,e,f) permeate quality. (T_f,i_ = 40 °C, T_p,i_ = 20 °C, Urine pH: ~6–7, COD: 2–6 g L^–1^, NH_4_–N: 0.1–0.2 g L^–1^, E .coli: < LOD; 1.8 wt% (Faeces/Urine) pH: ~7–9, COD: 7–15 g L^–1^, NH_4_-N: 0.1–1.3 g L^–1^, E. coli: ~ 4–8 log_10_ CFU mL^−1^).

Each configuration exhibited a rise in flux with increased feed temperature (Fig. 5) due to the exponential relationship between feed vapour pressure and temperature [44]. Interestingly, the total flux for VMD was found to be much lower than either AGMD or DCMD at each temperature studied. This is in contrast with most other comparative examples where the trend in flux is typically in the order VMD > DCMD > AGMD [21], [22], [50]. However, these examples are limited to aqueous salt solutions exhibiting a much different composition to the concentrated blackwater studied in this work. Visual inspection of the membranes after the experiments clearly indicates a higher degree of membrane surface fouling within the VMD experiments at the entrance to the module when compared to either AGMD or DCMD (Fig. 6) despite the vacuum being evenly distributed across the module from multiple vacuum ports, highlighting the need to tailor module design to a specific configuration. As the same module was used across configurations it would be expected that DCMD would exhibit a higher total flux than AGMD due to the additional resistance imposed to mass transfer by the stagnant air gap. It is therefore unexpected that both configurations exhibit very similar fluxes (Δ < 0.8 kg m^–2^h^−1^). Eykens et al. [23] demonstrated that for laboratory scale modules, DCMD exhibits a greater flux than AGMD; however, when performed at a pilot scale the inverse was true. This was attributed to the markedly increased temperature polarisation which occurs in a scaled up module which is exacerbated further by the increased conductive heat transfer in DCMD compared to AGMD [23]. However, the relatively small module size used (path length = 0.14 m) and low temperature polarisation measured across the DCMD module (≤6 °C) in this work means it is unlikely this would be the main cause for the similar observed fluxes. Additionally, when the feed is solely urine (Fig. 7a, b), the configurations also exhibit a similar total flux for a 0.1 μm PTFE membrane whereas the fluxes differ markedly at higher nominal membrane pore sizes. Therefore, it is unlikely the similar flux is caused by additional resistance imposed by a fouling layer arising from suspended solids present in concentrated blackwater. These results indicate that the membrane properties such as pore size, porosity and tortuosity are limiting mass transfer. For desalination, Eykens et al. [40] suggests a pore size of 0.3 μm results in an optimum trade-off between flux and wetting potential for DCMD and AGMD. A smaller pore size is required for VMD though to account for the greater hydrostatic pressure difference across the membrane [40]. The presence of organics in this work however will greatly impact the wetting potential of the feed and therefore alter these productivity-selectivity trade-off assumptions.

It can be seen that although each configuration can reduce organic and nitrogen content by several orders of magnitude, specific trade-offs in the control of volatile rejection and process productivity is observed between configurations. Vacuum membrane distillation provides enhanced selectivity towards water over ammonia, compared to AGMD and DCMD. This is offset though by the reduced productivity, caused by membrane surface fouling, increased energy input required to maintain the vacuum on the permeate side (60 W used in this work) and the difficulty in downscaling high vacuum pumps to the appropriate process scale. The increased complexity and cost required from the use of multiple stages for efficient latent heat recovery in VMD also negates its application in a low resource setting [51]. Although AGMD and DCMD exhibited similar water fluxes, the decreased selectivity of DCMD at lower feed temperatures would be disadvantageous as an intermittent or unstable thermal energy source (e.g. feed temperature ≤ 30 °C) may dramatically effect volatile rejection for DCMD, particularly ammonia. Contrastingly for AGMD, a consistent 60 – 80% rejection (dependent on solution pH) should be achieved. This greater degree of flexibility towards operating conditions lends itself to a low resource setting where a consistent and stable supply of thermal energy may not be available (e.g. solar, or faecal biomass combustion). Additionally, AGMD is the simplest process for latent heat recovery (dependent on system scale) as internal heat recovery in the module can be conducted where the feed is used as coolant to recover latent heat, so the process can be conducted off a single liquid pump [52]. DCMD on the other hand requires at least two pumps due to the separate feed and cooling solution and an additional external heat exchangers for heat recovery whilst VMD requires a liquid feed pump, vacuum pump and large external condenser to operate [52].

### 3.2 Faecal contamination reduces separation performance of MD but can be mitigated with precise membrane characteristics

Although for desalination using AGMD and DCMD an optimum membrane pore size of 0.3 μm [40] has been suggested to achieve a reasonable trade-off between salt retention and water flux, the markedly different solution chemistry of concentrated blackwater (consisting solely of urine and faeces) compared to seawater will greatly impact these assumptions of membrane pore size on separation performance. To investigate the impact of the constituents of concentrated blackwater, AGMD of urine was initially studied and compared to that of DCMD, which has received previous attention in this regard [34], [35], [37], [53]. The total flux generally increased for larger membrane pore sizes with both DCMD and AGMD (Fig. 7a, b), the effect being more pronounced for DCMD (Fig. 7a) due to the reduced resistance to mass transfer of this configuration. Although other membrane properties such as thickness and porosity (Table 1) will also effect mass transfer, the general increase in flux (Fig. 7a, b) is mostly attributable to the increasing pore sized membranes providing a decreased resistance to mass transfer [40]. This combined with the high rejection of COD (>98%) by both DCMD and AGMD up to a membrane pore size of 5 μm indicates that urine has a low wetting potential in both processes despite the low surface tension exhibited [36].

Both DCMD and AGMD exhibited a high degree of rejection of COD and ammonia from urine to produce a permeate with concentrations of COD: 22–60 mg L^–1^ and NH_4_-N: 9–32 mg L^–1^ (Urine: COD: 2000–6000 mg L^–1^, NH_4_-N: 100–200 mg L^–1^). Even at the larger membrane pore sizes studied (Fig. 7d, e) this is close to alignment of the European requirements for discharge from urban wastewater treatment plants (91/271/EEC COD: <125 mg L^-1^, Total Nitrogen: <15 mg N L^-1^) [8]. The permeate COD concentration at each membrane pore size is well below 100 mg L^–1^ as the majority of the organic fraction of urine is composed of non-volatile organics such as urea (~49% by weight total solutes), creatinine, and various organic acids [54]. Absolute COD concentration within the permeate was generally dictated by the concentration within the feed as opposed to relating to the nominal membrane pore size used. This indicates membrane wetting was not apparent, as this would cause non-volatile COD breakthrough into the permeate and a higher permeate concentration, particularly at the larger membrane pore sizes. Ammonia rejection is also ensured due to the pH of fresh urine being stable between ~6 and 7 over the course of the experiment (<6h) resulting in 99.5% of ammoniacal nitrogen present as the non-volatile ammonium ion (pH 6.5, 40 °C, NH_4_^+^ p*K*a = 8.8 [42]).

**Fig. 8:**
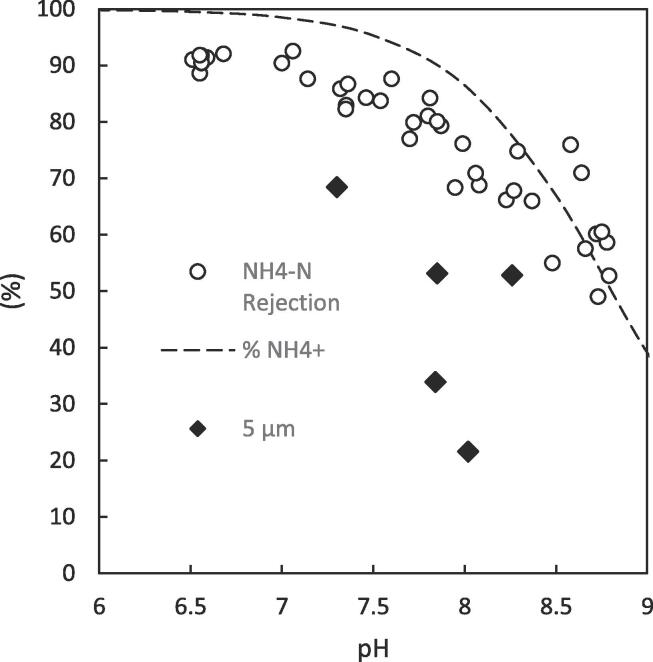
Collation of data for the rejection of ammoniacal nitrogen by AGMD at various faecal concentrations and membrane pore sizes. The data for the membrane of 5 μm pore size has been highlighted due to its deviation from the %NH_4_^+^ ratio as a result of membrane wetting. (T_f,i_ = 40 °C, T_p,i_ = 20 °C).

In contrast the introduction of faecal matter into the feed for AGMD resulted in markedly reduced permeate quality at higher nominal membrane pore sizes (Fig. 7f). This drastically increased total flux from 6.7 to 12.5 kg m^–2^h^−1^ and permeate COD concentration from 99 mg L^–1^ to 4265 mg L^–1^ for the 5 µm pore sized PTFE membrane. This reiterates the significant detrimental effect faecal matter has on the degree of membrane wetting in MD. Kamranvand et al. showed that the presence of faecal matter within urine reduces the overall surface tension of the solution [36], which would reduce the required transmembrane pressure to induce pore wetting, resulting in the observed phenomenon. However, for the smaller pore sizes (0.1, 0.45, 1 μm) the organic concentration within the permeate (82.4–110 mg L^–1^) was still below EU regulation for discharge under the urban wastewater treatment directive (<125 mg L^–1^) [8]. The ammoniacal nitrogen concentration in the permeate is also higher than for urine with the introduction of faecal matter (Fig. 7f). Although a degree of this will be due to membrane wetting of the larger pore sized membranes (particularly the 5 μm), ammonia rejection is still mainly determined by the feed pH (Fig. 8) and the ammonia-ammonium vapour liquid equilibrium. Whilst the membranes in this study exhibit only subtle variation in the membrane properties between pore sizes (porosity, thickness etc., Table 1), Volpin et al. showed that of a PTFE and PVDF membrane with the same nominal pore size (0.22 μm), the thicker PTFE membrane exhibited an enhanced water flux over ammonia compared to the thinner PVDF membrane [55]. This indicates that further subtle tuning of the ammonia selectivity could be achieved for membrane distillation through the appropriate selection of membrane material properties such as pore size, porosity, thickness, polymer material etc.

### 3.3 Air gap membrane distillation demonstrates recovery of high quality pathogen free water from concentrated blackwater

**Fig. 9:**
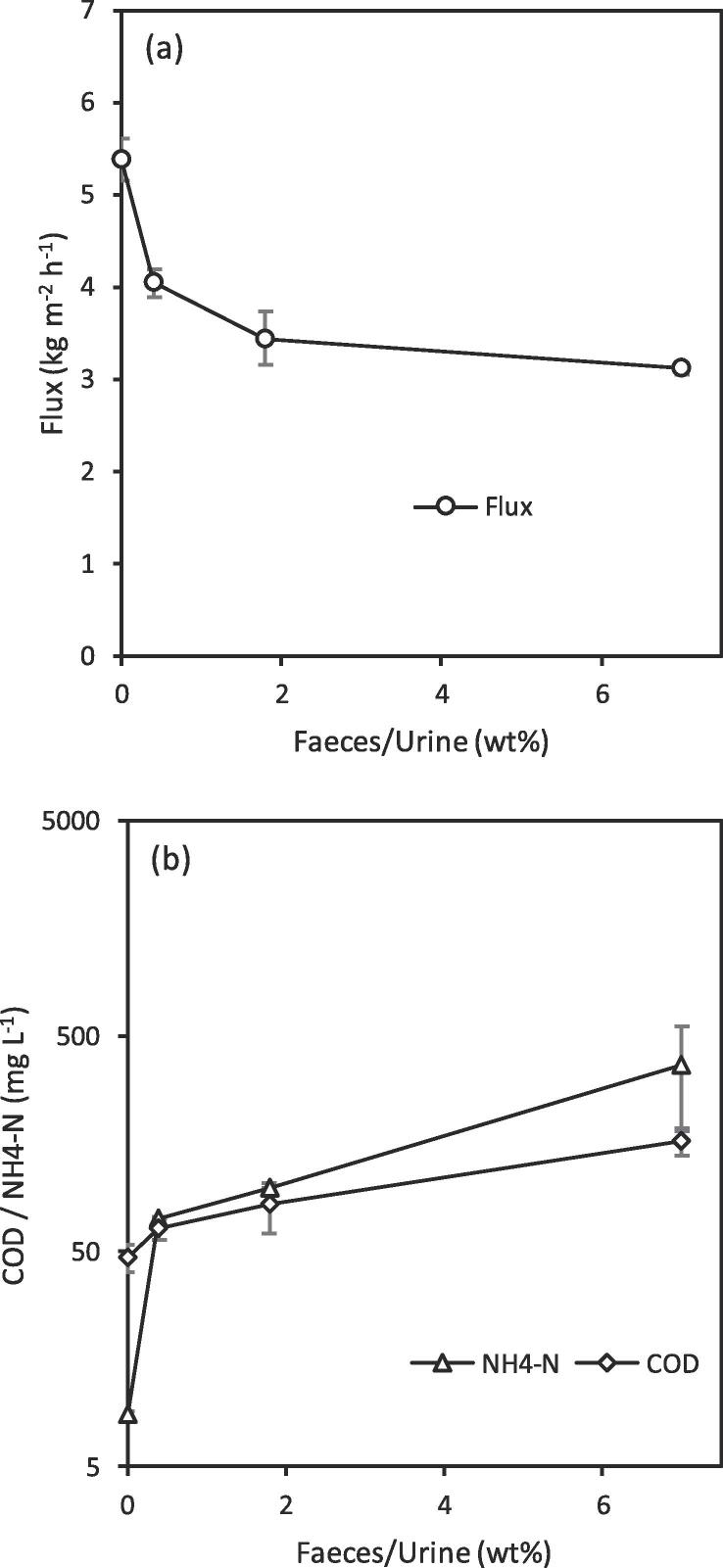
AGMD for water recovery from faecally contaminated urine: impact of the faecal contamination on (a) flux, (b) ammoniacal nitrogen and COD concentration in permeate (e). (0.1 μm PTFE membrane, T_f,i_ = 40 °C, T_p,i_ = 20 °C, Feed pH: ~6–9 COD: 2–15 g L^–1^ NH_4_-N: 0.1–3.5 g L^–1^ E .coli: ~LOD–8 log_10_ CFU mL^−1^).

Due to its detrimental impact on permeate quality, the concentration of faeces within concentrated blackwater was probed to determine the process robustness of AGMD and identify the critical ratio at which it can be operated whilst still producing a water quality meeting regulatory alignment for discharge. As faecal concentration may vary in concentrated blackwater from variations in the use of flush water and the levels of human defecation, the effect of varying faecal concentrations up to a 7 wt% (Faeces/Urine) ratio was investigated. This concentration is equivalent to the average ratio from human production with no dilution [10] and the homogenised sample represents the highest degree of faecal contamination that could possibly be delivered to a liquid treatment pathway. The increased organic loading and suspended solids from an increased concentration of faecal matter is known to detrimentally affect the total flux in membrane distillation [36]. This was demonstrated by the reduction in total flux through the 0.1 μm PTFE membrane when faecal contamination was increased (Fig. 9a). At the highest faecal loading (7 wt% (Faeces/Urine)) however, the total flux is only reduced by half compared to urine demonstrating a degree of resilience of process productivity to increasing faecal contamination.

The absolute concentration of COD within the permeate increased from 47 mg L^-1^ to 163 mg L^–1^ when the faecal contamination was increased from 0 to 7 wt% (Faeces/Urine). Despite the large amount of faecal contamination, a high rejection of ≥ 98% was observed for each concentration. Although the upper limit of this concentration is slightly greater than the discharge requirement of 125 mg L^–1^ COD set out by the European Urban Wastewater Treatment Directive (1991) [8], the reduction in COD load by ~14.8 g L^–1^ is exceptional for a single stage wastewater treatment separation. Likewise, the introduction of faecal matter (0–7 wt% (Faeces/Urine)) greatly increased the absolute concentration of ammoniacal nitrogen in the permeate from 8.7 mg L^-1^ to 367 mg L^-1^ (Fig. 9b). This nevertheless represents a remarkable reduction in ammoniacal nitrogen concentration of up to 874 mg L^–1^ when compared to the feed. Although much greater than the threshold of <15 mg N L^–1^ required by European legislation for discharge from urban wastewater treatment (91/271/EEC) [8], this absolute standard accounts for the effects of dilution by infiltration of stormwater, rainwater and greywater. The current scenario treats a feedwater >40 times more concentrated than traditional sewage and a more viable N load per capita is therefore much higher. This is underpinned within the ISO standard for non-sewered sanitation [7], which was devised to increase market penetration of such technology in low income countries through standardizing the required treatment potential of, and instilling consumer confidence in, new technologies. This standard requires a 70% reduction in total nitrogen in recognition of the much more concentrated feed and dilution of recovered water once released into the environment, a reduction that AGMD is close to consistently reaching (50–91% NH_4_-N). In addition, rejection of ammonia is still strictly related to the liquid phase ammonia-ammonium equilibrium (Fig. 8) defined by the solution pH. This indicates that even at high organic loadings minimal wetting of the membrane pores has occurred and demonstrates a degree of resilience within the separation process towards an increased faecal load.

**Fig. 10:**
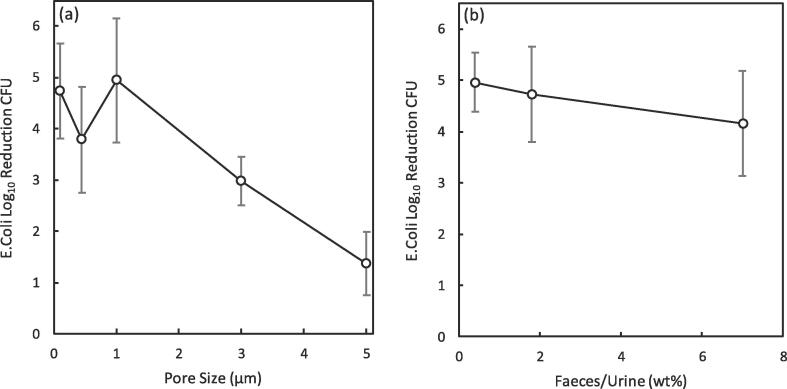
Reduction of E. Coli by AGMD for water recovery from faecally contaminated urine: (a) impact of the pore size and (b) faecal contamination. T_f,I_ = 40 °C, T_p,i_ = 20 °C (a) 1.8 wt% (Faeces/Urine) Feed pH: ~7–9, COD: 7–15 g L^–1^, NH_4_-N: 0.1–1.3 g L^–1^, E. coli: ~ 4–8 log_10_ CFU mL^−1^; (b) 0.1 μm PTFE membrane, , Feed pH: ~6–9, COD: 2–15 g L^–1^, NH_4_–N: 0.1–3.5 g L^–1^, E. coli: <1–8 log_10_ CFU mL^−1^).

As shown in Fig. 10a, E*. coli* were removed by the smaller pore sized membranes (≤1 μm), by a combination of the liquid-vapour interface and size exclusion due to *E. col*i being rod shaped with average sizes ~ 1 × 3 μm (w × l) [56]. At larger pore sizes (≥3 μm) though, higher concentrations of *E. coli* are observed within the permeate (≥3 log_10_ CFU mL^−1^) as they are able to permeate through the larger liquid bridged pores arising from wetting; the wetting mechanism being evidenced by the sharp increase in permeate COD (Fig. 7f). With increasing faecal concentration in the feed, the observed log_10_ *E. coli* reduction was high for the 0.1 μm membrane (Fig. 10b) ranging between ~ 3 – 6 and being comparable to other conventional disinfection processes such as chlorination (2–6), ozonation (2–6) and ultraviolet (2–4) treatment [57]. At lower faecal loadings (1.8 wt%, 0.4 wt% (Faeces/Urine)) effluent concentrations of ~ 0.5 log_10_ CFU mL^−1^ (LOD: −1 log_10_ CFU mL^−1^) were observed below the standard of ≤ 1 log_10_ faecal coliforms mL^−1^ set out for unrestricted agricultural reuse by the WHO [6]. However, when the faecal load was increased to 7 wt% the absolute values of *E. Coli* within the permeate were higher (≤1.2 log_10_ CFU mL^−1^) and slightly over the standard. Although size exclusion of *E. Coli* will still apply in this scenario, as there will be a distribution in pathogen size, greater quantities in the feed will result in the presence of more smaller cells which are able to penetrate the small membrane pore size and therefore explain the greater absolute value observed in the permeate. Therefore, the average faecal loading would most likely need to be controlled below 1.8 wt% (Faeces/Urine) during continuous operation. The high degree of separation within a single barrier for COD, ammoniacal nitrogen and pathogens by AGMD from a concentrated blackwater with a faecal concentration at the highest attainable limit evidences the process robustness and its promise as technology for inclusion within an integrated system for producing high quality pathogen free water from concentrated black water.

## 4 Conclusions

AGMD, DCMD and VMD have been investigated for water recovery from concentrated blackwater in the context of decentralised sanitation. Each configuration can produce high quality water but selectivity of the volatile fraction is dependent upon the applied configuration. VMD provided a high degree of separation towards volatiles, particularly ammonia at high feed temperatures (>50 °C), but suffered from severe membrane fouling and therefore a markedly reduced total flux. DCMD demonstrated high rejection of volatiles at higher temperatures (>50 °C) but at lower temperatures (<40 °C) exhibited very poor volatile rejection particularly for ammonia. AGMD provided a consistent separation of COD (98% ± 0.25) across the temperature range studied and ammoniacal nitrogen rejection was solely dependent upon the feed liquid phase equilibrium at all feed temperatures and therefore demonstrated the most robust separation across variable operating conditions.. The wetting potential of urine and concentrated blackwater in AGMD was probed further by varying the membrane pore size where a small nominal pore size (0.1 μm) provided high resistance to wetting at a trade-off of introducing a greater resistance to mass transfer. Under extreme conditions with a feed representative of the highest possible ratio of faeces to urine possible from human production, AGMD facilitated a single stage barrier for high removal of COD and pathogens close to current international regulations for discharge or unrestricted agricultural reuse (Permeate max: COD ≤ 163 mg L^–1^; *E. Col*i ≤ 1.2 log_10_ CFU mL^−1^; NH_4_-N ≤ 367 mg L^–1^; Required: COD < 125 mg L^–1^, *E. Coli* < 1 log_10_ CFU mL^−1^; Total N < 15 mg L^–1^ [5], [6]). In practical context these values represent a worst case scenario of produced water quality from AGMD applied in decentralised sanitation. Typically, either source separation or post flush treatment will limit the contamination of urine by faecal matter within the liquid phase of a decentralised sanitation system with a feed much more representative of urine, which AGMD could consistently meet discharge standards from [58]. Additionally, current discharge and reuse standards account for the effects of dilution by stormwater, rainwater and greywater meaning the reported separation provides a much more remarkable performance than current treatment technologies due to it reducing pollutant concentrations by up to 40 times more than realised in traditional blackwater treatment. Additionally, further long-term studies will be required with more detailed membrane surface analysis to truly understand the wetting and fouling phenomenon occurring within the membrane distillation process. Despite this, the current study demonstrates the enormous potential of AGMD to provide an important separation for facilitation of high removal of COD, ammoniacal nitrogen and pathogens from concentrated blackwater. for decentralised sanitation utilising low-grade waste heat. The additional potential for internal latent heat recovery within the module and process simplification through utilising a single liquid pump additionally identified AGMD as the most suitable configuration for decentralised sanitation utilising low-grade waste heat.

## CRediT authorship contribution statement

**C.J. Davey:** Conceptualization, Data curation, Formal analysis, Investigation, Methodology, Writing - original draft. **P. Liu:** Formal analysis, Investigation, Methodology. **F. Kamranvand:** Data curation, Investigation, Methodology. **L. Williams:** Funding acquisition. **Y. Jiang:** Funding acquisition. **A. Parker:** Funding acquisition. **S. Tyrrel:** Project administration, Resources, Writing - review & editing, Funding acquisition. **E.J. McAdam:** Conceptualization, Formal analysis, Methodology, Project administration, Resources, Writing - review & editing, Funding acquisition.

## Declaration of Competing Interest

The authors declare that they have no known competing financial interests or personal relationships that could have appeared to influence the work reported in this paper.
